# Long non-coding RNA PART1 predicts a poor prognosis and promotes the malignant progression of pancreatic cancer by sponging miR-122

**DOI:** 10.1186/s12957-021-02232-3

**Published:** 2021-04-17

**Authors:** Xibao Hu, Lei Zhang, Jingjing Tian, Junhong Ma

**Affiliations:** 1 Department of Digestive Medicine, First Teaching Hospital of Tianjin University of Traditional Chinese Medicine, No. 314, Anshanxi Road, Nankai District, Tianjin, 300193 China; 2 Department of Chinese Medicine, Geriatric Hospital of Tianjin Municipal Civil Affairs Bureau, Tianjin, 300100 China; 3 Department of Digestive Medicine, Tianjin Nankai Hospital, Tianjin, 300100 China; 4 Department of Gastrointestinal Surgery, Tianjin Nankai Hospital, Tianjin, 300100 China

**Keywords:** PART1, Pancreatic cancer, Progression, miR-122, Sponge

## Abstract

**Background and objectives:**

Long non-coding RNA (lncRNA) prostate androgen-regulated transcript 1 (PART1) was previously shown to exert an oncogenic role in several human cancers. However, whether PART1 is associated with the malignant progression of pancreatic cancer remains unclear. In the current study, we aimed to identify the role and potential mechanism of PART1 in pancreatic cancer.

**Methods:**

qRT-PCR was applied to detect PART1 expression in 45 cases of pancreatic cancer patients. The chi-square test was performed to assess the association between PART1 expression and clinicopathologic features, and Kaplan-Meier method was applied to evaluate overall survival. In vitro CCK-8, transwell invasion, and flow cytometry assays were applied to detect the effects of PART1 on cell proliferation, invasion, and apoptosis, respectively. Luciferase reporter and RNA immunoprecipitation assays were used to identify the regulatory mechanism between PART1 and miR-122.

**Results:**

PART1 expression was upregulated in pancreatic cancer tissues and cell lines. High PART1 expression was closely correlated with tumor size, T classification, clinical stage, and vascular invasion, and predicted a poor overall survival. PART1 knockdown significantly suppressed cell proliferation and invasion abilities of pancreatic cancer but promoted cell apoptosis. PART1 was found to serve as a molecular sponge of miR-122, and miR-122 inhibition partially reversed the inhibitory phenotypes of PART1 knockdown on pancreatic cancer cells.

**Conclusions:**

PART1 promotes the malignant progression of pancreatic cancer by sponging miR-122. The PART1/miR-122 axis might be a promising target for anticancer therapy in patients with pancreatic cancer.

## Background

Pancreatic cancer is one of the most highly malignant tumors with rapid growth and early metastasis and is the sixth leading cause of cancer-related death in China [1]. Although great improvements in therapeutic strategies, such as pancreatectomy, radiotherapy, and chemotherapy, have been developed in the last decades, the 5-year overall survival rate of patients with pancreatic cancer remains lower than 5% due to the high rate of recurrence and metastasis [2, 3]. Similar to other human malignancies, pancreatic cancer progression is the result of a complex, multi-step process associated with a large number of genetic changes [4], and its exact molecular mechanism underlying progression remains elusive. Hence, it is urgent to develop novel therapeutic targets for the treatment of pancreatic cancer.

Long non-coding RNAs (lncRNAs) are a novel class of RNA transcripts of greater than 200 nucleotides with lacking protein-coding potential [5]. LncRNAs have been shown to be important regulators in a wide range of physiological and pathological processes including carcinogenesis, immune response, inflammation, and oxidative stress [6–8]. Growing evidence suggested that dysregulation of lncRNAs exhibited as an oncogenic or tumor-suppressive activity in cancer [9, 10]. For example, in colon cancer, Liu et al. reported that lncRNA ZEB2-AS1 promoted cell proliferation and inhibited cell apoptosis via regulation of miR-143/bcl-2 axis [11]. In osteosarcoma, Ye et al. suggested that lncRNA FER1L4 induced cell apoptosis and suppressed epithelial-mesenchymal transition via a competing endogenous RNA (ceRNA) of miR-18a-5p to upregulate SOCS5 expression [12]. In lung cancer, Zou et al. found that lncRNA OECC promoted cell proliferation and metastasis via PI3K/Akt/mTOR signaling pathway [13]. Therefore, identification of lncRNAs involved in pancreatic cancer progression might help yield novel therapeutic targets.

Prostate androgen-regulated transcript 1 (PART1), also known as NCRNA00206, is located on human chromosome 5q12.1, which consists of 2495 nucleotides with 4 exons. Originally, by using cDNA microarrays, Lin et al. found that PART1 was a novel human prostate-specific androgen-regulated gene [14]. Subsequently, some studies showed that PART1 was frequently overexpressed in cancerous prostatic tissues [15, 16]. Afterwards, the oncogenic function of PART1 had been validated in several types of human cancer including non-small cell lung cancer, colorectal cancer, and bladder cancer [17–19]. Nevertheless, its role in pancreatic cancer is largely unidentified. Therefore, the purpose of this study was to investigate the impacts of PART1 on the proliferation, invasion, and apoptosis of pancreatic cancer cells, as well as elucidate the relevant mechanism of this action. Our findings suggested that PART1 promoted the malignant progression of pancreatic cancer by sponging miR-122.

## Materials and methods

### Patients and clinical specimens

Forty-five samples of fresh cancer tissues and para-tumor normal tissues (located > 2 cm from the tumors) were obtained from pancreatic cancer patients who were either receiving a pancreatectomy or undergoing other palliative surgical operations at Tianjin Nankai Hospital. These tissues were estimated and confirmed through pathological review by independent histopathologists. All samples were snap-frozen with liquid nitrogen and stored at −80 °C until processing. None of the patients received chemotherapy and/or radiotherapy before surgery. The clinical characteristics of the specimens are shown in Table 1. Informed consent was obtained from the patients before sample collection. The study was approved by the committees for the ethical review of research at Tianjin Nankai Hospital.

**Table 1:** The PART1 expression and its association with clinical characteristics of patients with pancreatic cancer

Characteristics	Patients (No.)	PART1 level		*χ*^2^	*P* value
		Low	High		
Age (years)				1.289	0.256
< 60	16	10	6		
≥ 60	29	13	16		
Gender				1.412	0.235
Male	31	14	17		
Female	14	9	5		
Tumor size (cm)				8.213	0.004
< 3	13	11	2		
≥ 3	32	12	20		
Differentiation				0.657	0.720
Well	11	5	6		
Moderate	17	10	7		
Poor	17	8	9		
T classification				13.096	0.001
T1	8	5	3		
T2	22	16	6		
T3	15	2	13		
Lymph node metastasis				1.585	0.208
No	12	8	4		
Yes	33	15	18		
Vascular invasion				9.911	0.02
No	21	16	5		
Yes	24	7	17		
Clinical stage				11.428	0.003
I	9	8	1		
II	16	10	6		
III	20	5	15		

### Cell line and cell culture

The established human pancreatic cancer cell lines AsPC-1 (RRID: CVCL_0152), Panc-1 (RRID: CVCL_0480), SW1990 (RRID: CVCL_1723), and BxPC-3 (RRID: CVCL_0186), and a normal human pancreatic ductal epithelial line (HPDE6c7, RRID: CVCL_0P38) were purchased from the Institute of Cell Biology, Chinese Academy of Sciences (Shanghai, China). All the cells were propagated in a humidified 37°C incubator containing 5% CO_2_ with RPMI-1640 medium (Thermo Fisher Scientific, Waltham, MA, USA), 10% FBS (Gibco, NY, USA), 100 U/ml penicillin G, and 100 μg/mL streptomycin.

### Cell transfection

The small hairpin RNA of PART1 (sh_PART1) and shRNA negative control (sh_NC) was designed and synthesized by RiboBio Co., LTD (Guangzhou, China). The miR-122 inhibitor and inhibitor control were obtained at Invitrogen Corporation (Carlsbad, CA, USA). AsPC-1 and BxPC-3 cell lines were cultured in 6-well plates for 24 h. Subsequently, the cells were transfected with 100 pmol of sh_PART1 or sh_NC and 150 pmol of miR-122 inhibitor or inhibitor control by using Lipofectamine 2000 reagent (Thermo Fisher Scientific, Waltham, MA, USA) according to the manufacturer’s protocol. Forty-eight hours after transfection, the cells were collected and processed for further assays.

### Quantitative real-time PCR (qRT-PCR)

Total RNA was extracted from tissues and cultured cells with TRIzol reagent (Thermo Fisher Scientific, Waltham, MA, USA), according to the manufacturer’s protocol. The RNA was reversely transcribed into complementary DNA by using PrimeScript RT Reagent Kit (Takara, Dalian, China) with random primers according to the instruction. qRT-PCR was performed with the SuperReal PreMix Plus SYBR Green (TIANGEN, Beijing, China) on an ABI PRISM 7900HT (Applied Biosystems, Foster City, CA, USA) with the following conditions: 95°C for 10 min, followed by 40 amplification cycles of 98°C for 10s and 60°C for 45 s. The expression level of gene was normalized internally by using the CT value of the housekeeping gene GAPDH and U6, and was calculated with the 2^−ΔΔCt^ method. The primers were as follows: PART1 forward: 5′-CAATAAGGCAGAAGAAGGTG-3′, reverse: 5′-GGAGAATCTGAAGTCCCAAG-3′; GAPDH forward: 5′-CCTGCCTCTACTGGCGCTGC-3′, reverse: 5′-GCAGTGGGGACACGGAAGGC-3′; miR-122 forward: 5′-TATTCGCACTGGATACGACACAAAC-3′, reverse: 5′-GCCCGTGGAGTGTGACAATGGT-3′; U6 forward: 5′-CTCGCTTCGGCAGCACA-3′, reverse: 5′-AACGCTTCACGAATTTGCGT-3′. Each experiment was performed in triplicate and repeated three times.

### Cell counting kit-8 (CCK-8) assay

Cell proliferation was assessed by using a CCK-8 kit (Dojindo, Shanghai, China), according to the instruction. The transfected cells were adjusted to 4 × 10^4^ cells/well and seeded in 96-well plates, and treated with 12 μl of CCK-8 solution at 0, 24, 48, and 72 h after cell incubation. The optical density (OD) value of each well at 450 nm was measured by using a microplate reader (BioTek, Winooski, VT, USA).

### Cell invasion assay

Cell invasion was evaluated by using matrigel transwell membrane filter inserts (Corning Costar, Lowell, MA, USA) in 24-well tissue culture plates. The transfected cells with serum-free RPMI-1640 were seeded on the upper chamber of transwell filters. RPMI-1640 containing 20% FBS was added to the lower chamber and then incubated for 24 h. Cells in the upper side of the filter were removed, and the cells across the membrane were fixed with 4% formaldehyde at 37°C for 30 min and stained with Crystal Violet (TIANGEN) for 20 min at 37°C.

### Flow cytometry analysis

Flow cytometry was performed to evaluate cell apoptosis by using a Annexin V-FITC apoptosis detection kit (BD Biosciences, San Jose, CA, USA), according to the manufacturer’s protocol. The transfected cells were cultured for 48 h, then washed three times with cold PBS, and resuspended with 1 × binding buffer at a concentration of 1 × 10^6^ cells/ml. After being stained with 5 μl of FITC-Annexin V for 30 min and 3 μl of propidium iodide (PI) for 20 min at 37°C in the dark, the rate of apoptotic cells was analyzed by using a FACSCalibur FACS flow cytometer (BD Biosciences).

### Western blotting analysis

The cultured cells were collected and lysed by using RIPA reagent with phosphatase inhibitors and protein phosphatase inhibitors (BD Biosciences) at 4°C. Equivalent amounts (30 μg) of protein were electrophoresed on 12% SDS-polyacrylamide gel and transferred to PVDF membranes (Cell Signaling Technology, Denver, MA, USA). Then, the membranes were incubated with primary antibodies of Bcl-2 (Abcam Cat# ab182858, RRID: AB_2715467), Bax (Abcam Cat# ab32503, RRID: AB_725631), and GAPDH (Abcam Cat# ab9485, RRID:AB_307275) at 4°C overnight. After incubating with anti-rabbit HRP-conjugated secondary antibody (Abcam Cat# ab6721, RRID: AB_955447) at 37°C for 2 h, the protein bands were observed by using an Image Quant LAS 4000 mini (GE Healthcare, Waukesha, USA) and quantified by using an Image Pro Plus Software for Windows, version 6.0, Bethesda, MD, USA (www.xrayscan.com).

### Luciferase reporter assay

The PART1 wild-type (Wt) and mutant-type (Mut) fragments contained miR-122 binding sites that were synthesized and inserted into pGL3 vector (Promega Corporation, Madison, WI, USA) for getting the PART1-Wt and PART1-Mut reporter plasmid, respectively. Then, 0.5 μg of the reporter plasmids was transfected in combination with 50 pmol of miR-122 inhibitor or inhibitor control by using lipofectamine 2000 reagent according to the manufacturer’s protocol. Luciferase activity was measured 48 h after transfection by using the Dual Luciferase Reporter Assay System (Promega). The relative luciferase activity (Firefly) was normalized to pRL-TK activity (Renilla).

### RNA-binding protein immunoprecipitation (RIP) assay

The EZMagna RIP RNA-binding protein immunoprecipitation kit (Millipore, Billerica, MA, USA) was used for detecting the direct interaction between PART1 and miR-122. The cultured cells were lysed by using RNA lysis buffer, and then incubated with magnetic beads conjugated with human anti-Ago2 (Pierce, Rockford, IL, USA) and anti-IgG (Pierce) antibodies according to the manufacturer’s protocol. PART1 level in the precipitates was measured by using qRT-PCR.

### Statistical analysis

All experiments were conducted at least three times. Data were analyzed with the GraphPad Prism Software for Windows, version 5.0, San Diego, CA, USA (www.graphpad.com), and presented as the mean ± standard deviation (mean ± SD). The significance of the difference was analyzed by using Student’s *t*-test between two groups or one-way analysis of variance with Bonferroni post-test between multiple groups. Value of *P* < 0.05 was considered to be statistically significant.

## Results

### PART1 is highly expressed in pancreatic cancer tissues and cell lines

**Fig. 1:**
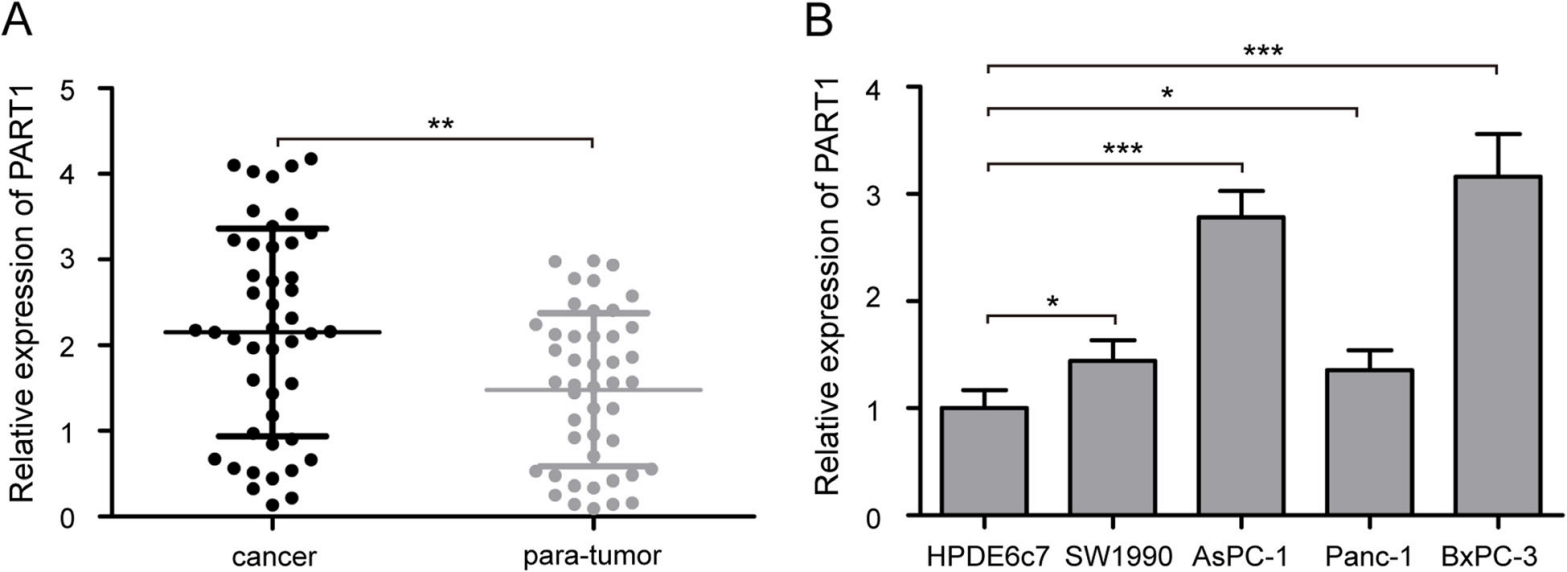
The expression level of PART1 in pancreatic cancer. **a** The expression of PART1 in cancer tissues and para-tumor normal tissues from 45 cases of patients with pancreatic cancer was determined by using qRT-PCR, the relative PART1 level was normalized to GAPDH. **b** The relative expression level of PART1 in four human pancreatic cancer cell lines (AsPC-1, Panc-1, SW1990, and BxPC-3) and a human pancreatic ductal epithelial cell line HPDE6c7 was shown. *P < 0.05, **P < 0.01, and ***P < 0.001

In order to assess the role of PART1 in pancreatic cancer, we performed qRT-PCR to detect its expression in 45 cases of pancreatic cancer patients, and found that the expression level of PART1 in cancer tissues was higher than that in para-tumor normal tissues (Fig. 1a, *P* < 0.01). Compared with HPDE6c7 cell line, all pancreatic cancer cell lines (AsPC-1, Panc-1, SW1990, and BxPC-3) showed increased PART1 expression (Fig. 1b, *P* < 0.05). Besides, among these pancreatic cancer cells, AsPC-1 and BxPC-3 cells had a higher PART1 expression than Panc-1 and SW1990 cells and were chosen for further experiments.

### High PART1 expression correlates with malignant clinicopathologic features and predicts a poor overall survival

**Fig. 2:**
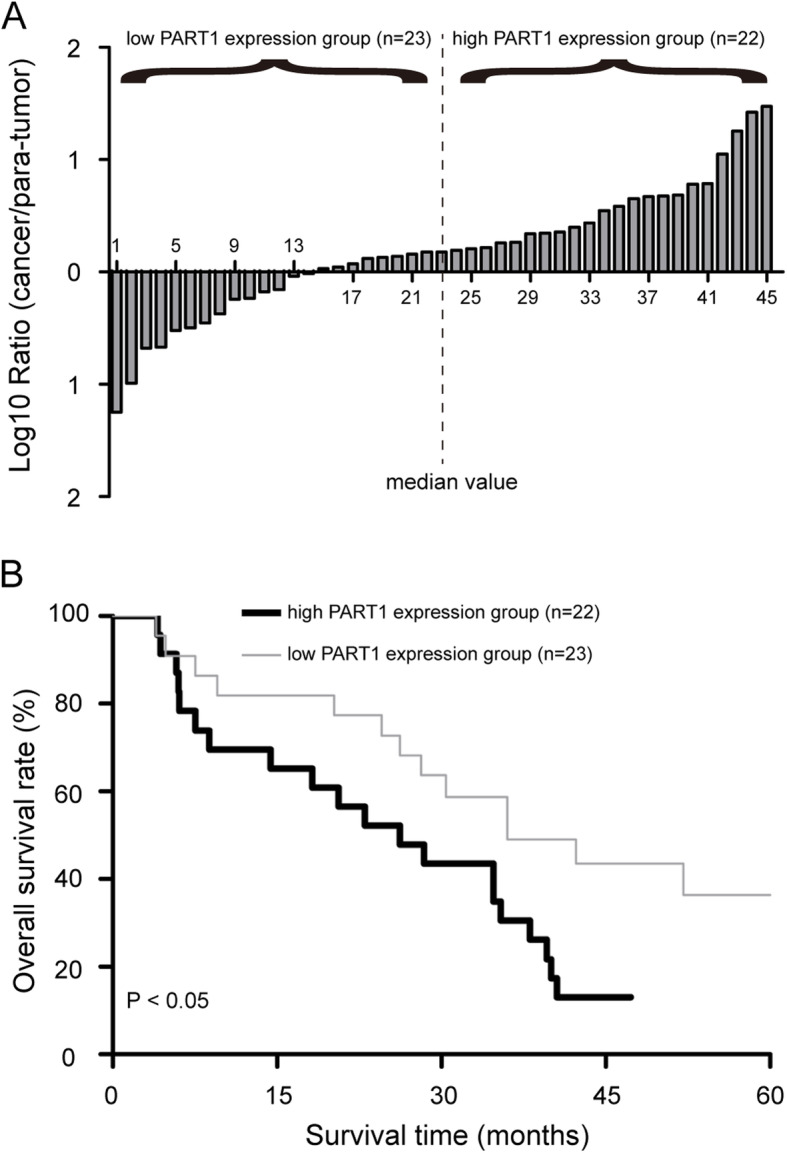
High PART1 expression predicted a poor prognosis of patients with pancreatic cancer. **a** Forty-five cases of patients were divided into low PART1 expression group (*n* = 23) and high PART1 expression group (*n* = 22) based on median value of PART1 expression in cancer tissues. **b** Kaplan-Meier curves for overall survival in the low versus high PART1 expression group from pancreatic cancer patients

We next evaluated whether PART1 expression correlated with the clinical outcomes in patients with pancreatic cancer. As shown in Fig. 2a, 45 cases of patients were divided into two groups based on median value of PART1 expression in cancer tissues, with 23 cases in low PART1 expression group and 22 cases in high PART1 expression group. By statistical analyses, high PART1 expression was shown to be correlated with tumor size, T classification, clinical stage and vascular invasion (Table 1, *P* < 0.05). No statistical correlation with age, gender, differentiation, and lymph node metastasis was observed (*P* > 0.05). Kaplan-Meier analysis revealed that the 5-year overall survival of high PART1 expression group was significantly shorter than that in low PART1 expression group (Fig. 2b, *P* < 0.05). These results indicated that high PART1 expression might be involved in the progression of pancreatic cancer.

### Knockdown of PART1 suppresses pancreatic cancer cell proliferation and invasion abilities

**Fig. 3:**
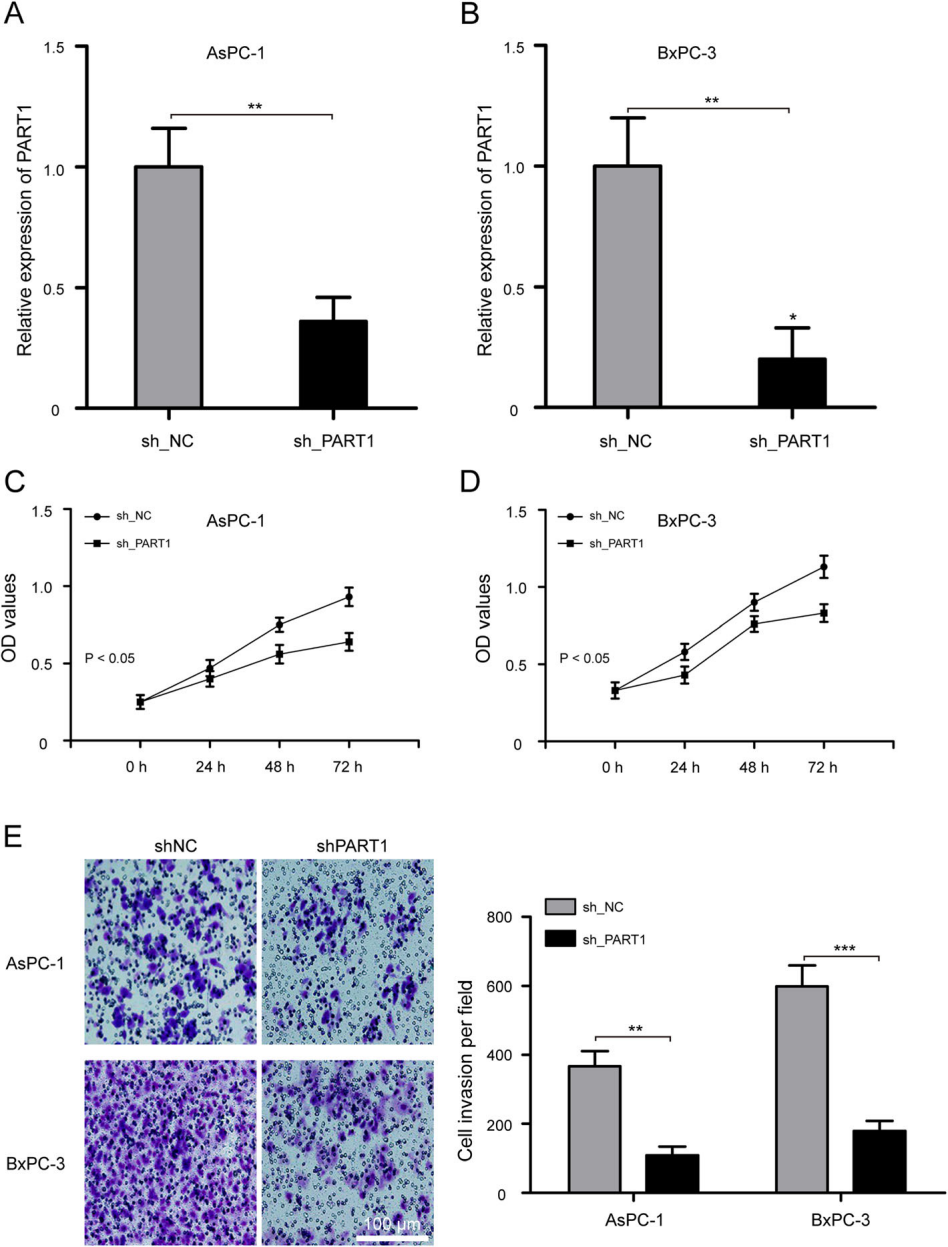
Knockdown of PART1 suppresses pancreatic cancer cell proliferation and invasion abilities. **a** qRT-PCR analysis of PART1 expression level in AsPC-1 cells after treatment with sh_PART1 and sh_NC. **b** BxPC-3 cells transfected with sh_PART1 significantly inhibited PART1 expression compared to the cells transfected with sh_NC. **c** The proliferation of AsPC-1 cells transfected with sh_PART1 and sh_NC was detected by CCK-8 assay. **d** PART1 knockdown significantly repressed cell proliferation of BxPC-3 cells. **e** Transwell invasion assay was performed to explore the effect of PART1 knockdown on cell invasion ability of AsPC-1 and BxPC-3 cells. **P < 0.01 and ***P < 0.001

Based on the highly expressed PART1 in pancreatic cancer, we investigated the role of PART1 in AsPC-1 and BxPC-3 cells with sh_PART1 and sh_NC. qRT-PCR confirmed that both the AsPC-1 and BxPC-3 cells transfected with sh_PART1 significantly inhibited PART1 expression compared to the cells transfected with sh_NC (Fig. 3a and b, *P* < 0.01). To further investigate whether PART1 knockdown could influence the proliferation of pancreatic cancer cells, in vitro CCK-8 assay was performed. As shown in Fig. 3c, the proliferation ability of AsPC-1 cells decreased significantly after transfection with sh_PART1 for 24, 48, and 72 h (*P* < 0.05). Similarly, BxPC-3 cells also showed lower proliferation ability following PART1 knockdown (Fig. 3d, *P* < 0.05). Subsequently, we applied transwell invasion assay to explore the effect of PART1 on cell invasion. Results revealed that cell invasion ability was depressed in AsPC-1 and BxPC-3 cells after being transfected with sh_PART1 (Fig. 3e, *P* < 0.01). These results demonstrated that PART1 promoted the proliferation and invasion of pancreatic cancer cells.

### Knockdown of PART1 promotes pancreatic cancer cell apoptosis

**Fig. 4:**
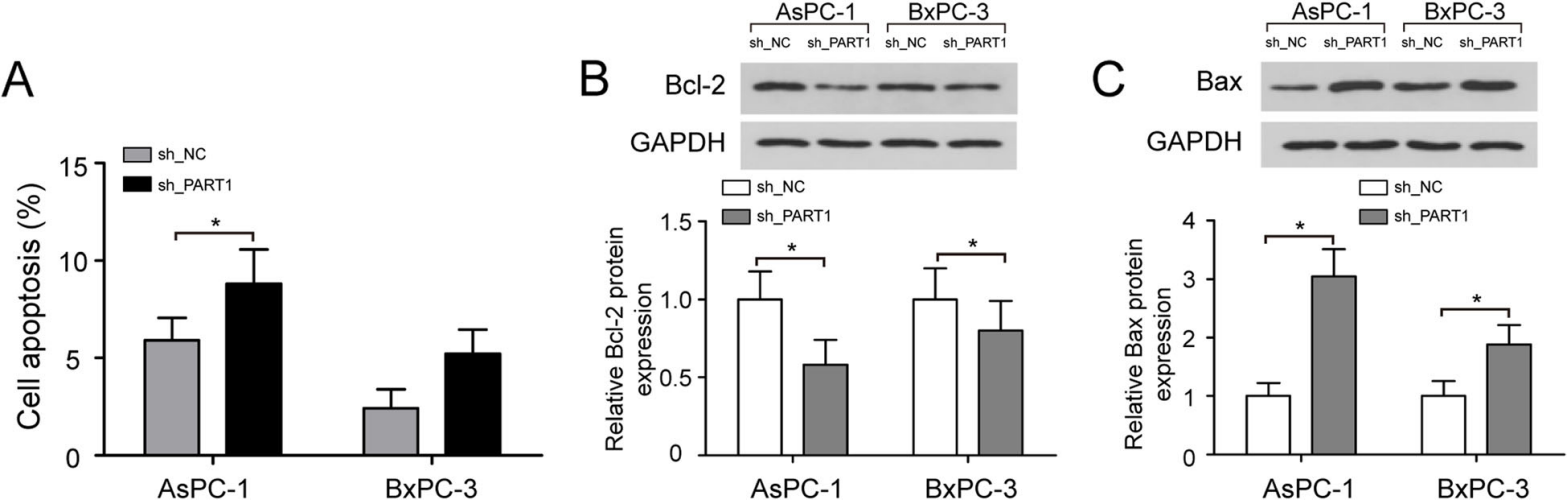
Knockdown of PART1 promotes pancreatic cancer cell apoptosis. **a** Flow cytometry analysis of the rate of apoptotic cells in AsPC-1 and BxPC-3 cells after transfected with sh_PART1 and sh_NC. **b** Western blotting analysis of Bcl-2 protein expression in AsPC-1 and BxPC-3 cells after PART1 knockdown. **c** Silencing of PART1 increased Bax protein expression in AsPC-1 and BxPC-3 cells. *P < 0.05

We further determined whether PART1 inhibition could also impact the apoptosis of pancreatic cancer cells; flow cytometry analysis was performed. Results showed that the rate of apoptotic cells was significantly increased in AsPC-1 and BxPC-3 cells after being transfected with sh_PART1 compared with the cells treated with sh_NC (Fig. 4a, *P* < 0.05). Additionally, we measured the effect of PART1 knockdown on Bcl-2 and Bax protein expression by using Western blotting analysis. As shown in Fig. 4b and c, silencing of PART1 decreased the protein expression level of Bcl-2 in AsPC-1 and BxPC-3 cells, but increased Bax protein expression (*P* < 0.05). These findings suggested that PART1 suppressed pancreatic cancer cell apoptosis.

### PART1 functions as a ceRNA by sponging miR-122

**Fig. 5:**
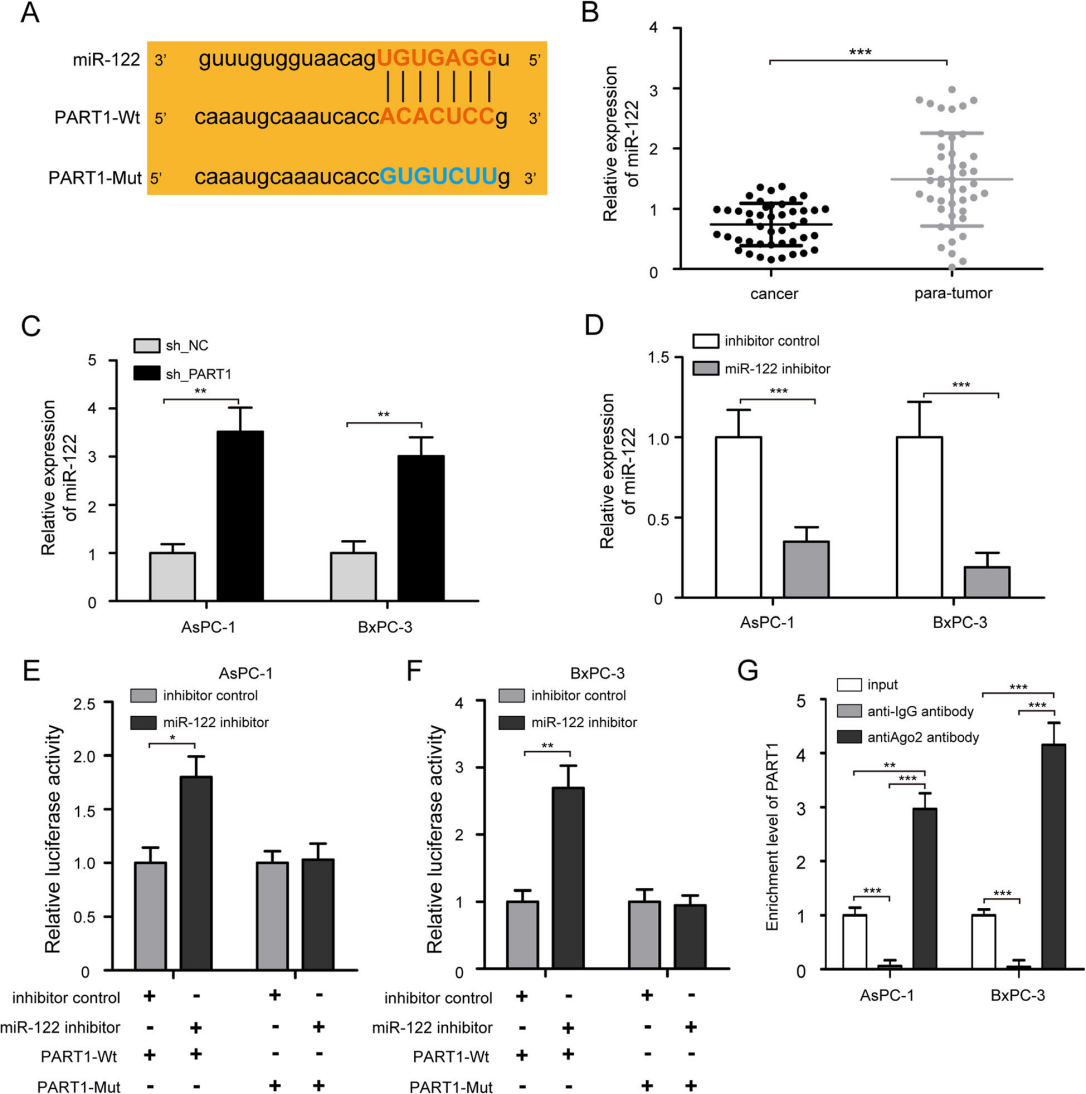
PART1 functions as a ceRNA by sponging miR-122. **a** The putative miR-122 binding sites of the wild-type (Wt) and mutant (Mut) sequences of PART1. **b** qRT-PCR analysis of miR-122 expression in cancer tissues (*n* = 45) and para-tumor normal tissues (*n* =45). **c** The expression of miR-122 in AsPC-1 and BxPC-3 cells was increased by PART1 knockdown. **d** qRT-PCR confirmed miR-122 expression in AsPC-1 and BxPC-3 cell after being transfected with miR-122 inhibitor and inhibitor control. **e**, **f** The PART1-Wt or PART1-Mut reporter vector was cotransfected with miR-122 inhibitor and inhibitor control into AsPC-1 and BxPC-3 cells, and the relative luciferase activity was detected. **g** RIP assay with anti-Ago2 and anti-IgG antibodies was performed in AsPC-1 and BxPC-3 cells, and the coprecipitated RNA was used to quantify PART1 expression. *P < 0.05, **P < 0.01, and ***P < 0.001

In order to elucidate the potential mechanism of PART1 involved in the progression of pancreatic cancer, the online predictive tool of lncRNAs (http://starbase.sysu.edu.cn/starbase2/index.php) was used for bioinformatics analysis and found that PART1 (NR_028509.1) contained putative binding sites for miR-122 (Fig. 5a). Compared with para-tumor normal tissues, the expression level of miR-122 was downregulated in cancer tissues (Fig. 5b, *P* < 0.001). We further analyzed the effect of PART1 on miR-122 expression in AsPC-1 and BxPC-3 cells, and found that PART1 knockdown upregulated the expression level of miR-122 (Fig. 5c, *P* < 0.01). Subsequently, a luciferase reporter assay was used to confirm the binding sites between PART1 and miR-122. Transfection of miR-122 inhibitor decreased miR-122 expression in AsPC-1 and BxPC-3 cells in comparison with the cell treated with inhibitor control (Fig. 5d, *P* < 0.001). Expectedly, the results of the luciferase reporter assay revealed that miR-122 inhibitor could elevate the relative luciferase activity of the PART1-Wt reporter vector in AsPC-1 and BxPC-3 cells, whereas this effect was abolished when the cells co-transfected with miR-122 inhibitor and PART1-Mut reporter vector (Fig. 5e, f, *P* < 0.05). Simultaneously, RIP assay revealed that PART1 expression was enriched by anti-Ago2 antibody in comparison with anti-IgG antibody in AsPC-1 and BxPC-3 cells, indicating PART1 directly bound to miR-122 (Fig. 5g, *P* < 0.01). These results suggested that PART1 functioned as a ceRNA by sponging miR-122.

### PART1 function is partially mediated by sponging miR-122

**Fig. 6:**
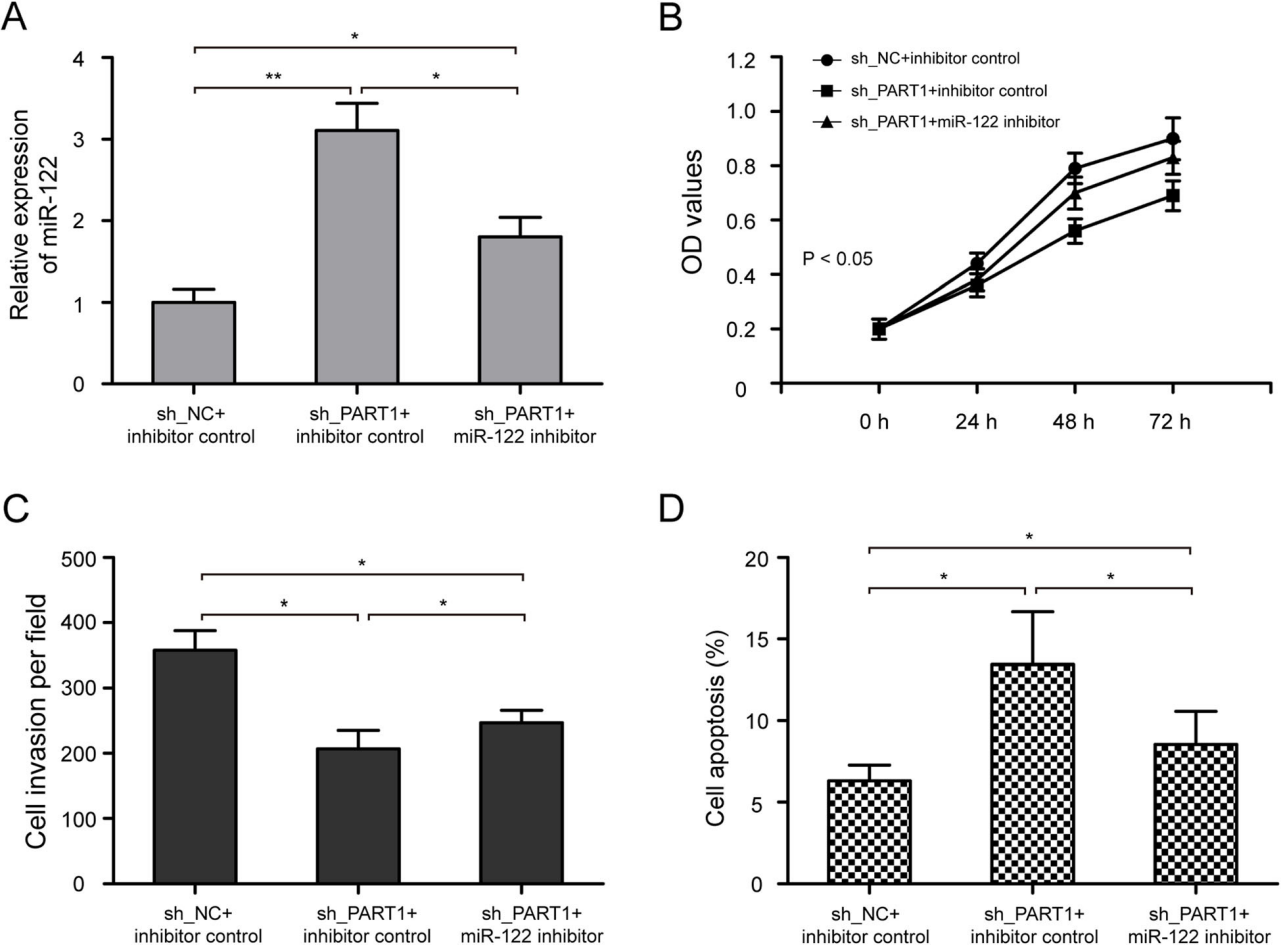
miR-122 inhibition partially reverses the inhibitory phenotypes of PART1 knockdown on pancreatic cancer cells. **a** The expression of miR-122 in PANC-1 cells transfected with sh_PART1, together with miR-122 inhibitor or inhibitor control was determined by qRT-PCR. **b** The cell proliferation ability of AsPC-1 cells in sh_NC + inhibitor control, sh_PART1 + inhibitor control, and sh_PART1 + miR-122 inhibitor groups was determined by CCK-8 assay. **c** miR-122 inhibitor could partially abrogate the inhibitory effect of PART1 knockdown on cell invasion in AsPC-1 cells. **d** miR-122 inhibitor could partially reverse the increased cell apoptotic rate in AsPC-1 cells caused by inhibiting PART1. *P < 0.05 and **P < 0.01

To explore whether PART1 exerted biological effect through miR-122, AsPC-1 cells were transfected with sh_PART1, together with miR-122 inhibitor or inhibitor control. The qRT-PCR results revealed that miR-122 inhibitor could partially decrease miR-122 expression in AsPC-1 cells by sh_PART1 (Fig. 6a, *P* < 0.05). CCK-8 assay showed that miR-122 inhibitor could reverse the reduction of cell proliferation ability caused by PART1 knockdown in AsPC-1 cells (Fig. 6b, *P* < 0.05). The transwell invasion assay revealed that miR-122 inhibitor could partially abrogate the inhibitory effect of PART1 knockdown on cell invasion in AsPC-1 cells (Fig. 6c, *P* < 0.05). In addition, flow cytometry analysis showed that increased cell apoptotic rate in AsPC-1 cells caused by inhibiting PART1 was partially reversed by miR-122 inhibitor (Fig. 6d, *P* < 0.05). All the above results demonstrated that PART1 promoted the malignant progression of pancreatic cancer by sponging miR-122.

## Discussion

Increasing evidences has implied that lncRNAs expression is frequently altered in cancer tissues and cell lines, and may represent attractive therapeutic targets in pancreatic tumors [20, 21]. In fact, several lncRNAs have been reported to be involved in the progression of pancreatic cancer [22]. For example, lncRNA DLEU2 is an important promoter of pancreatic cancer progression by modulating cell proliferation and invasion [23]. Upregulation of lncRNA TUG1 regulates cell cycle, proliferation, and apoptosis of pancreatic cancer [24]. LINC00052 functions as a tumor suppressor through modulating miR-330-3p in pancreatic cancer [25]. In addition, lncRNA SUMO1P3 promotes cell proliferation, migration, and invasion of pancreatic cancer [26]. The roles of PART1 in the progression of several human cancers have been well documented in the previous investigations. Here, to our knowledge, this is the first research that confirmed that PART1 exerted an oncogenic function in the malignant progression of pancreatic cancer via acting as a sponge for miR-122.

As a novel identified lncRNA, PART1 has been reported to be a promising biomarker for prediction of survival and tumor recurrence in stages I–III non-small cell lung cancer [27]. Previous studies reported that PART1 expression was upregulated in non-small cell lung cancer, colorectal cancer, and bladder cancer [17–19]. Consistent with their study results, our experiment results showed that PART1 was highly expressed in pancreatic cancer tissues and cell lines. Furthermore, we observed that high PART1 expression was correlated with tumor size, T classification, clinical stage, and vascular invasion, and predicted a poor overall survival. These data implied that high PART1 expression might play critical roles in pancreatic cancer progression. However, PART1 expression was not related to lymph node metastasis; the possible explanation might be caused by the small size of the samples. Therefore, these data must be treated and interpreted with caution, and more clinical samples should be included to further confirm the clinical significance of PART1 in future studies.

According to the studies of Zhu et al. and Lou et al. [17, 28], interference of PART1 inhibited the progression of non-small cell lung cancer and colorectal cancer. Up to now, there was no report about the role of PART1 in pancreatic cancer. In our study, we used a synthetic sh_PART1 to target PART1 in pancreatic cancer cells. Functional assays showed that PART1 knockdown suppressed pancreatic cancer cell proliferation and invasion abilities in vitro. Our results verified that PART1 promoted cell proliferation and invasion in pancreatic cancer.

Previous research demonstrated that PART1 was associated with tumor cell apoptosis in cancer [19]. Sun et al. found that PART1 modulated toll-like receptor pathways to influence cell apoptosis in prostate cancer [29]. Zhou et al. found that inhibition of PART1 promoted colorectal cancer cell apoptosis [30]. Here, we transfected sh_PART1 into AsPC-1 and BxPC-3 cells and found that silencing of PART1 increased pancreatic cancer cell apoptosis. The Bcl-2 is an apoptosis-associated gene, and homodimer of Bcl-2 inhibits apoptosis [31]. Bax is a crucial component of mitochondrial membrane ion channels, and homodimer of Bax induces the formation of apoptotic bodies [32]. When the Bcl-2/Bax heterodimer is formed, Bax is able to inhibit the anti-apoptotic function of Bcl-2 resulting in the promotion of apoptosis. To better understand the mechanism of PART1 on pancreatic cancer cell apoptosis, we measured the effect of PART1 knockdown on Bcl-2 and Bax protein expression. Results showed that knockdown of PART1 decreased the protein expression level of Bcl-2 in AsPC-1 and BxPC-3 cells, but increased Bax protein expression. Consistent with the above reports, the findings of our study supported an oncogenic role for PART1 in pancreatic cancer.

The ceRNA hypothesis proposes that lncRNAs exert their function by sponging miRNAs [33]. To understand the mechanism of PART1-regulated pancreatic cancer progression, we searched public bioinformatics database and found PART1 might be a sponge of miR-122. miR-122 has been thought to be downregulated in a variety of human cancers. In terms of the function, Xu et al. demonstrated that miR-122 inhibited cell migration and invasion in gastric cancer by downregulation of DUSP4 [34]. Cui et al. reported that miR-122 suppressed proliferation and invasion of pancreatic cancer cells through targeting ALDOA [35]. Consistently, we found that miR-122 expression was decreased in pancreatic cancer tissues. Yet, the regulatory mechanism between PART1 on miR-122 remains largely elusive. Specifically, our research showed that PART1 knockdown could increase miR-122 expression. According to the dual-luciferase reporter and RIP assay results, PART1 functioned as a ceRNA by sponging miR-122. Moreover, miR-122 knockdown could partially reverse the inhibitory effects of PART1 silencing on cell proliferation, invasion, and apoptosis. Collectively, we deduced that PART1 could promote pancreatic cancer progression; its mechanism may be related to act as a sponge of miR-122. Similarly, work by Wang et al. has demonstrated that PART1 promoted breast cancer cell progression by sponging miR-4516 [36].

However, this study still had some deficiencies. The effects of PART1 and miR-122 on pancreatic cancer growth and metastasis in vivo should be identified. Because lncRNAs have more than just one targeted gene, other potential targets of PART1 should be investigated in future studies.

## Conclusion

PART1 is found to be upregulated in pancreatic cancer. PART1 promotes the malignant progression of pancreatic cancer by sponging miR-122. Thus, PART1/miR-122 axis might be a novel therapeutic target for pancreatic cancer treatment.

## Data Availability

The datasets during and/or analyzed during the current study are available from the corresponding author on reasonable request.
